# Molecular detection and genetic characterization of *Arcobacter butzleri* isolated from red-footed pet tortoises suspected for *Campylobacter* spp. from Grenada, West Indies

**DOI:** 10.1371/journal.pone.0230390

**Published:** 2020-03-16

**Authors:** Bhumika Sharma, Katelyn Thille, Vanessa Matthew Belmar, Roxanne Nicholas Thomas, Ravindra Nath Sharma

**Affiliations:** Department of Pathobiology, School of Veterinary Medicine, St. George’s University, Grand Anse, Grenada, West Indies; University of Campinas, BRAZIL

## Abstract

The aim of the study was to detect and genetically characterize *Arcobacter butzleri* in pet red-footed tortoises suspected for *Campylobacter* spp., using molecular techniques. A written consent from tortoise owners was obtained, after explaining the advantages of the research to tortoise owners of Grenada. Fecal samples were collected from 114 tortoises from five parishes of the country and cultured for *Campylobacter* spp. using selective culture techniques. *A*. *butzleri* was isolated from 4.39% of pet tortoises. Total thirteen isolates were obtained; all identified as *A*. *butzleri* by a universal and a species-specific Polymerase Chain Reaction (PCR) and direct sequencing. Genetic characterization of these isolates was performed based on Enterobacterial repetitive intergenic consensus PCR (ERIC-PCR) that generated eight different genetic fingerprints with a discriminatory power of 0.91. *Campylobacter* species were not detected molecularly in any of the culture-positive samples. This is the first report of infection of pet tortoises in Grenada, West Indies with *A*. *butzleri*. This study emphasizes on the risk of zoonotic transmission of *A*. *butzleri* by exotic pets, which is a serious concern for public health.

## Introduction

The genera *Arcobacter* and *Campylobacter* were initially included in the family *Campylobacteriacae* [1]. Later the genus *Arcobacter* was assigned to a new family *Arcobacteriacae* [2]. To date 27 species have been recognized within the genus *Arcobacter* [3], and 26 species, 2 provisional species, and 9 subspecies have been recognized within the genus *Campylobacter* [4]. *Campylobacter* and *Arcobacter* species are the most common re-emerging food-borne zoonoses worldwide [4–8]. *Campylobacter* spp. are found in warm-blooded animals, mostly observed in food animals (poultry, cattle, sheep, goats and pigs) [9]. Often, animals are asymptomatic carriers of the diseases. *Campylobacters* localize host intestinal tract and are shed in feces. Animal-meat gets contaminated from feces during unhygienic slaughter. *Campylobacter* is transmitted to humans from raw, uncooked, or contaminated meat especially poultry, as well as contaminated water and milk [10–15]. Direct contact with infected animal may also cause transmission to humans. Like *Campylobacter*, *Arcobacter* also infects various farm animals like cattle, chickens, pigs, sheep and ducks, causing abortions, mastitis and diarrhea [16–18]. *Arcobacter* has been isolated from humans and the clinical symptoms are like that of *Campylobacter* infections except that instead of bloody diarrhea, *Arcobacter* usually has a persistent watery diarrhea [19].

Morphologically it is very difficult to differentiate between cells of the genus *Arcobacter* to those of *Campylobacter*; both genera consist of Gram-negative rods, curved, S-shaped, or helical bacteria [20]. It has been observed in routine diagnostic laboratory that during the initial isolation, a combination of both *Campylobacter* and *Arcobacter* species can be present on cultured plates [21]. Differentiating *Arcobacter* species from those of *Campylobacter* may be difficult using phenotypic or biochemical methods [22].

Prevalence of *Campylobacter* and *Arcobacter* spp. in pet animals is not well documented. However, recent reports suggest that pathogens from both genera harbor companion pet animals [23–26]. Furthermore, some reports emphasize on the vast distribution and prevalence of *Campylobacter* and *Arcobacter* in host species not previously documented such as exotic pet animals [27–30]. The expanded host range of *Campylobacter* and *Arcobacter* now includes captive reptiles such as chelonians, lizards and snakes [2, 31–33]. Gilbert et al. [33] reported isolation of *Epsilonproteobacteria* from intestinal contents of reptiles (chelonians, snakes and lizards) from Netherlands and *A*. *butzleri* was one of the most commonly isolated bacteria among others.

In the Caribbean region, *Campylobacter* spp. have been reported from food animals, dogs and chickens from Trinidad [34] however, speciation of these isolates was not performed. Research conducted in Barbados [35] and Grenada [36–39], revealed the presence of *C*. *coli* and *C*. *jejuni* in food animals and pet dogs in highest frequencies. There is a paucity of information regarding *Arcobacter* species prevalence in vertebrates in the Caribbean region. There is only one published report from Trinidad in which the authors analyzed nest-sand and egg-shell samples of leatherback turtles and found *Arcobacter* species in less than 10% of the samples through culture and biochemical testing [40]. However, no molecular tests were performed to further characterize these species.

Red-footed tortoise (*Chelonoidis carbonaria*) is found on the island of Grenada and locals keep them as pets [41]. This species is mostly herbivorous, feeding on leaves, fruits and flower but they can also feed on carrion and feces [42].To the author’s knowledge, there are no published reports on the molecular detection of *Campylobacter* and/or *Arcobacter* species in turtles and tortoises in Grenada. However, based on *Campylobacter* prevalence reports from Grenada and the coprophagic-feeding behavior of red-footed tortoise, the authors suspected fecal-oral transmission of *Campylobacter* in red-footed tortoises. Thus, the objective of this study was to detect and genetically characterize bacterial species isolated from pet red-footed tortoises suspected for *Campylobacter* spp., using molecular techniques.

## Materials and methods

### Ethical approval

The research project was approved by the Institutional Animal Care and Use Committee (IACUC) of St. George’s University (IACUC 18009 23rd August 2018).

### Study site and consent of tortoise pet owners

Grenada is the southernmost country in the Caribbean Sea with an area of 348.5 km^2^. The country, with low hills, small trees and shrubs and tropical climate is a most suitable habitat for tortoises. The environmental temperature remains within a range of 20°C to 30°C. The country is divided into six parishes: Saint Patrick, Saint Mark, Saint Andrew, Saint John, Saint George and Saint David. The terrain throughout the country is similar. Tortoise owners were identified, with the assistance of Mr. Derek Thomas, Livestock officer, Veterinary Department, Ministry of Agriculture, Land, Fishery and Forest, Grenada, West Indies. The research plan was explained to the pet owners in detail, and a written consent was obtained from those who agreed to participate in the research. The number of samples collected from each parish is as follows: Saint Patrick *n = 17;* Saint Andrew *n = 22;* Saint John *n = 13;* Saint George *n = 52;* Saint David *n = 10*. No samples were available from Saint Mark.

### Collection of fecal samples

An island-wide random sampling method was adopted to meet the aim of the research on population of pet tortoises in Grenada. Sample size estimation was based on the formula given by Glenn [43] as: N = t^2^ (p) (1-p) /d^2^ where t = 1.96 (for a 95% confidence level); p = estimated prevalence of the condition and d = desired level of precision. Since the incidence of *Campylobacter* in tortoises in Grenada is not known, estimated incidence was taken between 5% to 10%, at a confidence interval of 5%. The formula gave a sample size ranging between 73 to 138. Demographic information on location of tortoises in different parishes, sex and age was taken at the time of sample collection. Fecal samples from 114 tortoises were collected using cloacal swabs in Cary Blair transport medium (Becton Dickson and Company, Maryland, USA) for a period of five months from November 2018 to March 2019. The swabs were transported on ice within 4 hours after collection to the microbiology laboratory of School of Veterinary Medicine and cultured the same day. For collection of feces on cloacal swabs, tortoises were placed on their backs on a hard surface and the cloaca was stimulated by gentle finger touch. The tortoise usually voided fresh feces and cloaca became soft.

### Bacterial culture

For bacterial culture, methodology described by Hariharan et al., [38] was followed. The cloacal-swab samples were plated on *Campylobacter* blood-free selective agar (CBF) containing charcoal, cefoperazone and amphotericin B supplement (Oxoid Ltd. Basingstoke, Hampshire, England). The plates were incubated at 42°C for 48 hours under microaerophilic conditions (5% oxygen, 10% carbon dioxide and 85% nitrogen) using Campy-gas generating pack (BBL, Becton Dickson and Co. Maryland, USA). Colonies were stained with Gram’s stain and examined under microscope. Positive colonies were sub-cultured on another CBF plate for isolation of pure cultures. To speciate the isolates, the cultures were subjected to biochemical tests including catalase and oxidase (BBL, Becton, Dickinson and Co., Sparks, MD, USA), and hippurate tests (Remel, Lennexa, KS, USA). Pure isolates were then transferred and stored in 10% skim milk in cryovials at -80°C [44] until the DNA extraction was performed. The above described culture methods were also performed on the reference strain *Campylobacter jejuni subsp*. *jejuni* ATCC® 33291^™^ (ATCC, Manassas, VA, USA) and used as a positive control for subsequent experiments.

### DNA extraction

Thirteen pure culture isolates (from five tortoise-fecal samples) were thawed at room temperature and then centrifuged at 300×g for 5 min. The supernatant was discarded, and the pellet was resuspended in 200μL of Phosphate Buffer Saline. Each sample tube was then processed for DNA isolation using DNeasy Blood and Tissue extraction Kit (Qiagen, Hilden, Germany) according to the manufacturer’s instructions. DNA from the reference strain *C*. *jejuni subsp*. *jejuni* ATCC® 33291^™^ (ATCC, Manassas, VA, USA) was extracted separately using the exact procedure mentioned above and was used as a positive control for PCR protocols. DNA purity and concentration was checked spectrophotometrically using NANODROP 2000 (Thermo Fisher Scientific, Waltham, Massachusetts, USA).

### Screening PCRs

Three different PCR protocols were performed to analyze the isolates molecularly. The sequences and origin of the three sets of primers used for gene amplification are indicated in Table 1. The initial identification of the isolates was based on the amplification of a ~450 bp *16S rRNA* gene fragment, targeting different bacterial species, using universal, degenerate primers [45]. These isolates were further characterized and differentiated based on a *Campylobacter* genus-specific and an *A*. *butzleri-*specific PCR [46, 47]. Each PCR amplification was carried out using a 25μl reaction mixture containing a final concentration of 1× Platinum Hot Start PCR Master Mix ((1.5 mM MgCl_2_; 0.2 mM dNTPs; 2 U of Taq DNA polymerase) Thermo Fisher Scientific, Waltham, MA USA)), 0.5 mM MgCl_2_, 0.5 μM of each primer, and 1μl (~7 ng to 30 ng) of DNA template. Nuclease-free water was used as a negative control and genomic DNA extracted from the reference strain *C*. *jejuni subsp*. *jejuni* ATCC® 33291^™^ (ATCC, Manassas, VA, USA) served as a positive control. The cycling conditions were as follows: initial denaturation at 95°C for 5 minutes followed by 40 cycles of denaturation at 95°C for 30 sec; annealing at 51°C for 1 minute (MD16S1-MD16S2 primer pair), 60°C for 1 minute (16s2F-16s4R primer pair), 55°C for 1 minute (16SArcobutzFw-16SArcobutzRv primer pair), and extension at 72°C for 1 minute, and a final 7 minutes extension at 72°C after the last cycle.

**Table 1:** Oligonucleotide primers used in this study.

Primer Name	Sequence (5´ to 3´)	Target gene	Target species	Amplicon size (bp)
**16s2F 16s4R**	CCTACGGRSGCAGCAG GGACTACCMGGGNTATCTAATCCKG	16S rRNA	Bacteria	~450
**MD16S1 MD16S2**	ATCTAATGGCTTAACCATTAAAC GGACGGTAACTAGTTTAGTATT	16S rRNA	*Campylobacter* genus	857
**16SArcobutzFw 16SArcobutzRv**	AGTTGTTGTGAGGCTCCAC GCAGACACTAATCTATCTCTAAATCA	16S rRNA	*A*. *butzleri*	203

Twenty-five microliters of the PCR products were subjected to electrophoresis with 1.5% agarose gel, stained with ethidium bromide, and photographed under gel documentation system (LabNet International Inc., Edison, New Jersey, USA). Amplicons were purified using QIAquick Gel Extract Kit (Qiagen, Hilden, Germany) following manufacturer’s instructions and sent for direct sequencing to the sequencing facility provided by Molecular Cloning Laboratories (South San Francisco, CA, USA).

### DNA fingerprinting

Enterobacterial repetitive intergenic consensus PCR (ERIC-PCR) was performed for the genetic characterization of the *A*. *butzleri* strains. The primers used for this protocol are ERIC1R (5´-ATGTAAGCTCCTGGGGATTCAC-3´) and ERIC2 (5´-AAGTAAGTGACTGGGGTGAGCG-3´) designed by Versalovic et al. [48]. Each ERIC-PCR amplification and cycling conditions were carried out based on the protocol described by Houf et al. [49]. Briefly, a 50μl reaction mixture containing a final concentration of 1× PCR reaction buffer, 0.5μM of the forward primer and the reverse primer, 0.2mM of the dNTPs, 4mM of MgCl_2_, 5U of the Platinum Taq DNA polymerase and ~5–25 ng/μl of template DNA was used for each reaction. Nuclease-free water was used as a negative control. The cycling conditions were as follows: initial denaturation at 94°C for 5 minutes followed by 40 cycles of 1 minute denaturation at 94°C, 1 minute of annealing at 25°C, and 2 minutes of extension at 72°C, and a final 5 minutes extension at 72°C after the last cycle.

### Analysis of ERIC-PCR products

Ten microliters of the PCR products were size separated by electrophoresis in ethidium bromide-stained 1.5% agarose gels with 1×Tris-acetate-EDTA buffer for 2.5 hours at 100 Volts. The DNA profiles were visualized by UV transillumination and photographed using gel documentation system (LabNet International Inc., Edison, New Jersey, USA). DNA patterns that differed in one or more DNA fragments were considered patterns that represented different types. To interpret the profiles of the fecal isolates, a software program GelJ [50] was used. GelJ uses the gel picture to generate a computer-based DNA band clustering matrix. Based on the presence or absence of a band, a binary matrix is marked with 1 (or +), or 0 (or -) respectively. This band clustering matrix is used to compute a similarity matrix using Dice coefficient [51], which expresses the similarity level between two DNA patterns. Based on the similarity matrix a dendrogram was constructed by using the unweighted pair group linkage analysis method (UPGAM). Numerical index of discrimination for ERIC-PCR was calculated by Simpson’s index of diversity [52] using the following formula:

$$D=1-\frac{1}{N\;(N-1)}\sum_{j=1}^{s}nj\;(nj-1),$$

where D = index of discriminatory power; N = the total number of strains in the sample population; nj = the number of strains belonging to the j^th^ type; and s = the total number of types defined.

## Results

### Bacterial culture and DNA extraction

Out of the 114 tortoises’ fecal samples, five (4.38%) showed colony-growth seen as little pinpoint translucent colonies, different from grayish non translucent colonies characteristic of *Campylobacter* spp. [53]. Sub-culturing of the five bacterial colonies gave 13 pure isolates. Gram’s-staining and microscopy revealed Gram-negative, curved bacilli with corkscrew-like motility. Isolates tested biochemically were found to be oxidase positive, catalase positive, and hippurate hydrolysis negative. The DNA concentration of these 13 isolates ranged between 7ng/μl to 30ng/μl. The DNA concentration of the reference strain *Campylobacter jejuni subsp*. *jejuni* ATCC® 33291^™^ (ATCC, Manassas, VA, USA), was 32 ng/μl.

### Screening PCRs

**Fig 1:**
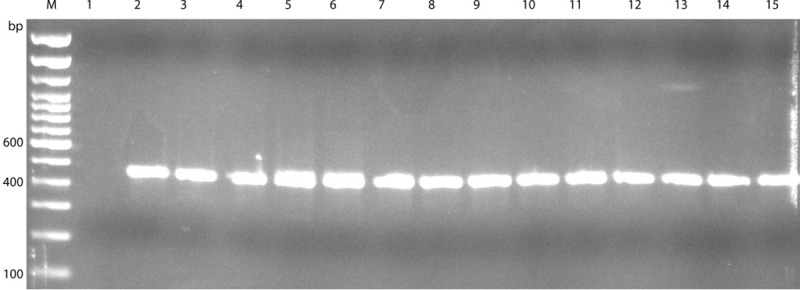
Gel photograph of PCR using degenerate, universal primer pair16s2F-16s4R. Lane M: Trackit 100bp ladder; Lane1: negative control (dH_2_O); Lane2: Reference strain (*C*. *jejuni subsp*. *jejuni* ATCC® 33291^™^; Lane3: T5; Lane4: T7; Lane5: T13.1; Lane6: T13.2; Lane7: T25.1; Lane8: T25.2; Lane9: T25.3; Lane10: T25.4; Lane11: T41.1; Lane12: T41.2; Lane13: T41.3; Lane14: T41.4; Lane15: T41.5.

The PCR run using degenerate, universal primer pair16s2F-16s4R, resulted in the amplification of an approximately 450 bp DNA fragment from all 13 isolates as well as the reference strain *C*. *jejuni subsp*. *jejuni* ATCC® 33291^™^ (ATCC, Manassas, VA, USA) (Fig 1).

This result suggested that the isolates were of bacterial origin which was confirmed by the sequencing results. All 13 sequences were compared to the sequence-database present in GenBank® using the Nucleotide Basic Local Alignment Search Tool of National Center for Biotechnology Information. The results from 16S rRNA gene sequences of all 13 sequences showed 99.5% to 100% homology to *A*. *butzleri* strain ED-1 (accession #CP041386.1). Partial sequences for 16S rRNA gene from 13 samples were submitted to GenBank® as recorded in Table 2.

**Table 2:** New *A*. *butzleri* sequences submitted to GenBank® database for a 400 bp 16S rRNA gene fragment.

Sample ID	Accession number	Percent similarity to *Arcobacter butzleri* strain ED-1 (accession #CP041386.1)
**T5**	MN731608	100
**T7**	MN731609	100
**T13.1**	MN731610	99.5
**T13.2**	MN731611	99.75
**T25.1**	MN731612	100
**T25.2**	MN731613	100
**T25.3**	MN731614	100
**T25.4**	MN731615	100
**T41.1**	MN731616	100
**T41.2**	MN731617	100
**T41.3**	MN731618	100
**T41.4**	MN731619	100
**T41.5**	MN731620	100

**Fig 2:**
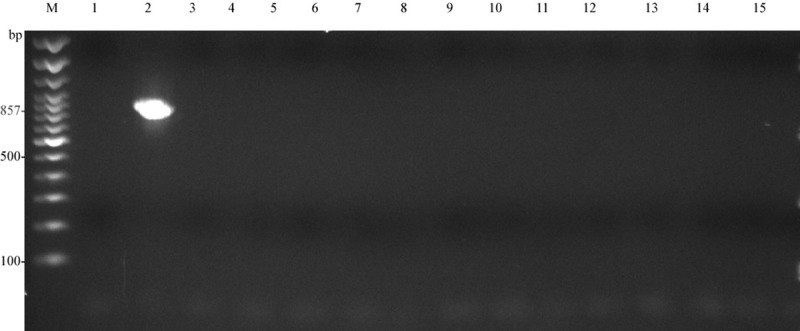
Gel photograph of PCR using *Campylobacter* genus-specific primer-pair MD16S1- MD16S2. LaneM: Trackit 100bp ladder; Lane1: negative control (dH_2_O); Lane2: Reference strain (*C*. *jejuni subsp*. *jejuni* ATCC^®^ 33291^™^; Lane3: T5; Lane4: T7; Lane5: T13.1; Lane6: T13.2; Lane7: T25.1; Lane8: T25.2; Lane9: T25.3; Lane10: T25.4; Lane11: T41.1; Lane12: T41.2; Lane13: T41.3; Lane14: T41.4; Lane15: T41.5.

**Fig 3:**
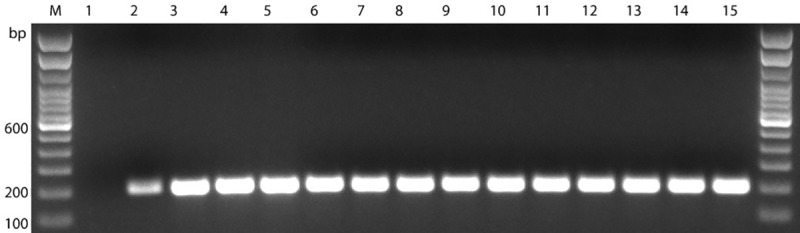
Gel photograph of PCR using *A*. *butzleri specific* primer pair 16SArcobutzFw-16SArcobutzRv. LaneM: Trackit 100bp ladder; Lane1: negative control (dH_2_O); Lane2: Reference strain (*C*. *jejuni subsp*. *jejuni* ATCC® 33291^™^; Lane3: T5; Lane4: T7; Lane5: T13.1; Lane6: T13.2; Lane7: T25.1; Lane8: T25.2; Lane9: T25.3; Lane10: T25.4; Lane11: T41.1; Lane12: T41.2; Lane13: T41.3; Lane14: T41.4; Lane15: T41.5.

Agarose gel electrophoresis for the *Campylobacter* genus-specific PCR, using primer pair MD16S1-MD16S2, did not result in amplification of the 857 bp fragment from any of the 13 isolates except the reference strain *C*. *jejuni subsp*. *jejuni* ATCC® 33291^™^ (ATCC, Manassas, VA, USA)(Fig 2). Agarose gel electrophoresis for the *A*. *butzleri-*specific PCR, using primer pair 16SArcobutzFw-16SArcobutzRv, resulted in the amplification of a 203 bp fragment from all 13 isolates as well as a faint band for the reference strain *C*. *jejuni subsp*. *jejuni* ATCC® 33291^™^ (ATCC, Manassas, VA, USA) (Fig 3).

### DNA fingerprinting and analysis of ERIC-PCR products

**Fig 4:**
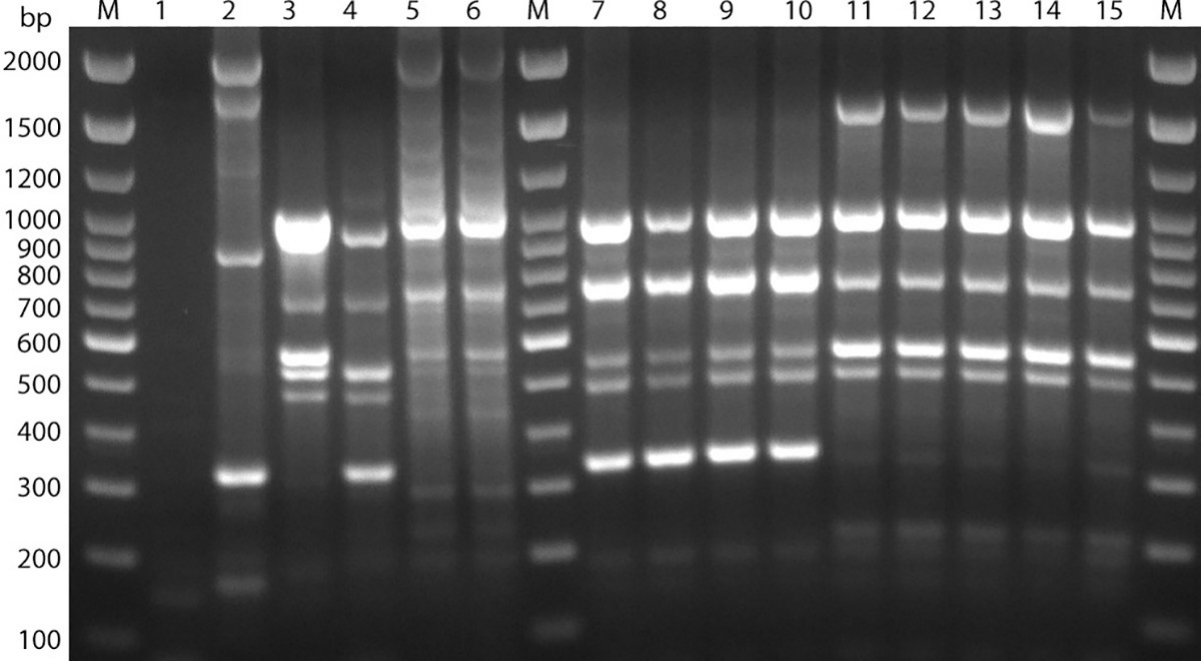
The ERIC profiles obtained for the 13 *A*. *butzleri* isolates. Lanes M: Trackit 100 bp DNA ladder; Lane1: negative control (dH_2_O); Lane2: Reference strain (*C*. *jejuni subsp*. *jejuni* ATCC® 33291^™^; Lane3: T5; Lane4: T7; Lane5: T13.1; Lane6: T13.2; Lane7: T25.1; Lane8: T25.2; Lane9: T25.3; Lane10: T25.4; Lane11: T41.1; Lane12: T41.2; Lane13: T41.3; Lane14: T41.4; Lane15: T41.5.

**Fig 5:**
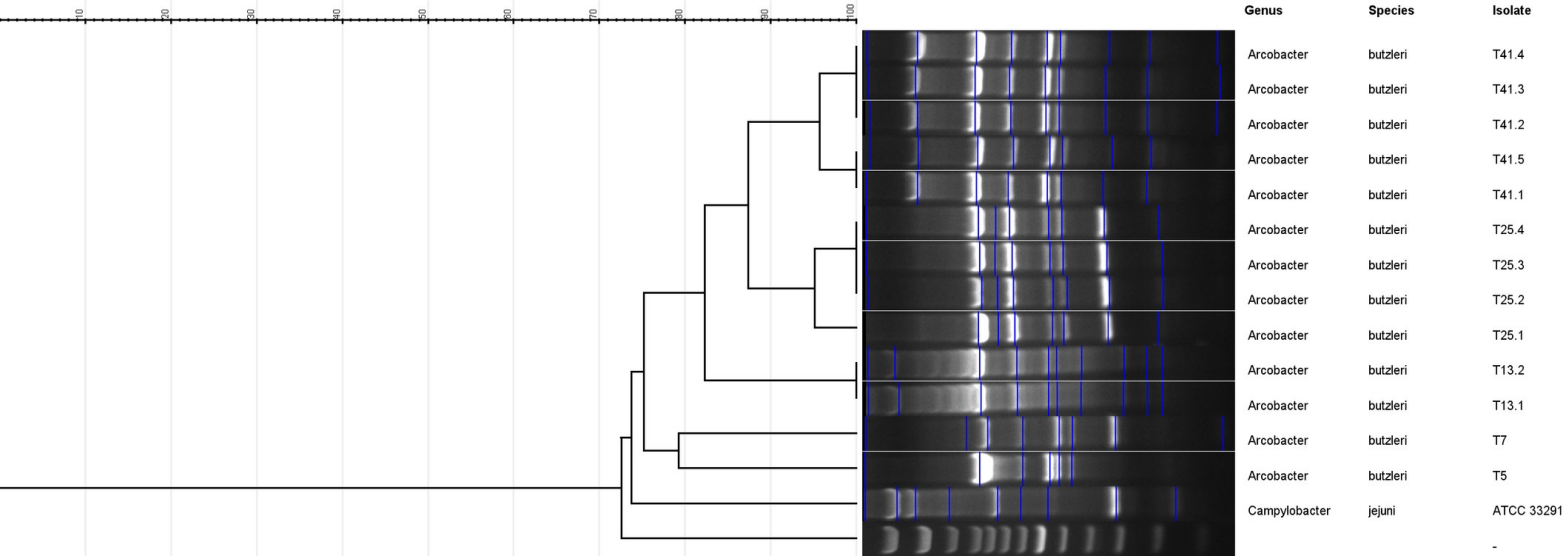
Dendrogram showing eight ERIC-types obtained for *A*. *butzleri* isolates.

ERIC sequences were found in all 13 *A*. *butzleri* isolates. Less variation was seen for the ERIC sequences with respect to the isolates of same fecal samples. ERIC-PCR resulted in 6–10 distinct fingerprints for *A*. *butzleri* isolates ranging between ~200 bp and ~2000 bp in length (Fig 4). Dendrogram analysis revealed that *A*. *butzleri* isolates from the same fecal samples mostly clustered together in the same group (Fig 5), and eight different ERIC patterns (ERIC-types) were observed for the 13 *A*. *butzleri* isolates (Table 2). The discriminatory power of ERIC-PCR was calculated to be 0.91 based on the Simpson’s index of diversity [52].

## Discussion

Since the authors were mainly interested in *Campylobacter* species, the culture plates were incubated at 42°C which is the optimal growth temperature for these species. *Arcobacte*r spp. can grow under aerobic, microaerobic or H_2_-enriched microaerobic conditions at lower temperatures ranging between 15°C and 37°C, while some strains can grow at temperature as high as 42°C [54]. During present investigation, colonies, morphologically resembling *Campylobacter* spp., were isolated. A total of 13 pure isolates were found after subculture of plates. All isolates tested biochemically, were found to be oxidase positive, catalase positive, and hippurate hydrolysis negative. These biochemical tests are highly nonspecific since both *Campylobacter* and *Arcobacter* species have the same reaction to these biochemicals [54]. Thus, it is difficult to differentiate *Arcobacter* and *Campylobacter* species based on phenotypic and biochemical methods [22]. This issue is addressed by Figueras et al [55], in a case report where a 26-years old male was diagnosed with acute gastroenteritis due *Campylobacter* initially based on stool-culture and biochemical testing of the isolates. A PCR-based molecular testing performed on these isolates later identified *Arcobacter cryaerophilus* as the causative species. In addition to this, reports by Collado et al. [56] and Boer et al [57] demonstrate that PCR-based molecular methods are more sensitive in detecting *Arcobacter* than culture methods. Various PCR protocols have been developed for the identification and confirmation of *Campylobacter* and *Arcobacter* [46, 58, 59]. During the current research, we performed three different sets of PCR experiments to accurately identify the isolated colonies. It is very surprising that none of the 13 isolates came positive via *Campylobacter* genus-specific PCR even though, morphologically, the colonies resembled *Campylobacter*. Only 4.39% of the samples (5/114) were culture positive but, all 13 isolates from these five positive samples were identified as *A*. *butzleri* by two different PCR sets. These results suggest that the culturing temperature (42°C), may have been too high for culturing potential *Campylobacter* and *Arcobacter* from chelonians with low preferred body temperatures. Further research using more stringent culture conditions for the growth of *Campylobacter* and *Arcobacter* is recommended.

ERIC-PCR based genotyping, resulted in bands ranging between 6–10 (~200-2000bp); all 13 isolates were typable (100%) and a total of eight ERIC-types were observed with a discriminatory power of 0.91. ERIC-PCR discriminatory powers of 0.91 indicated ERIC-PCR to be a highly desirable genotyping method since discriminatory powers above 0.90 are considered highly significant [52].

Among all *Arcobacter* spp., *A*. *butzleri* is considered an emerging food and water-borne pathogens [5, 60]. The bacterium causes illness in animals and humans. In animals, it causes gastroenteritis, and reproductive disorders, while in humans’ gastroenteritis is the main symptom. *A*. *butzleri* has been identified worldwide; in chickens and wastewater in Spain [61] in sheep-cheese in Italy [62]; in retail meat from (chicken, beef, pork and lamb) in Poland and Brazil [63, 64] in diarrheic and non-diarrheic humans in Canada [65]. The mode of transmission of *Arcobacter* spp. is poorly understood. However, it is suggested that transmission is through contaminated food of animal origin, water and vegetables [60, 66]. There is a paucity of information on the occurrence of *Arcobacter* spp. in reptiles. According to Gilbert et al 2014 [33], out of the study population of 417 captive reptiles, chelonians harbored *Campylobacter*, *Arcobacter* and *Helicobacter* spp. in highest frequencies, detected by culture isolation (37%) as well as PCR (82.5%) and *Arcobacter* species were the most commonly isolated bacteria among others. *A*. *butzleri* was isolated from 7.1% (11/154) of the chelonians which is higher than what we have observed in the current study. To this date, there is no published data on the molecular detection of *Arcobacte*r spp. from any of the sources (humans, animals, reptiles and food products) from Grenada and other Caribbean islands.

In the present research, we report isolation of *A*. *butzleri*, the most pathogenic member of *Arcobacter* genus. The incidence rate of *A*. *butzleri* was low (4.39%) in examined pet red footed tortoise from Grenada, compared to 7.1% reported by Gilbert et al [33]. This could be attributed to the sub-optimal culture conditions, nonetheless, these findings should be viewed as a serious concern for public health. As seen in this study and reported from some European countries [67] the number of reptiles kept as pets is increasing and so is the risk of diseases associated with these animals [68, 69]. There is also a report of increased farming of reptiles, mainly freshwater turtles for human consumption in some Asian countries [70]. Thus, reptilian-derived *A*. *butzleri* infections in humans should be considered a high-risk factor. It should also be noted here that apart from the normal feed-related transmission, the possibility of fecal-oral transmission is likely to occur, especially in captive animals particularly chelonians of the Testudinidae family since these species commonly display coprophagy [33]. With this concern for human health, pet owners should be informed of the danger of this pathogen and educated to prevent the transmission of infection to humans (pet owners, handlers and visitors to tortoise farms).

This is the first report of isolation and molecular detection of *A*. *butzleri*, from red-footed tortoises from Grenada, West Indies. *Arcobacter* is an emerging food-borne zoonotic pathogen and has been classified as a serious hazard to human health by International Commission on Microbiological Specifications for Foods (ICMSF). Isolation of *A*. *butzleri* from exotic pets like red-footed tortoises justify the possibility of fecal-oral transmission of this pathogen and could potentially lead to the transmission of this zoonosis to the owners, pet handlers or anyone in close proximity to these pets. Since morphological and biochemical reactions cannot differentiate between *Campylobacter* spp. and *Arcobacter* spp., PCR be employed to identify these two genera. In the present research molecular techniques (PCR) proved to be useful in identification and genetic characterization of *A*. *butzleri*.

## Supporting information

S1 Fig Raw gel photograph of PCR using degenerate, universal primer pair16s2F-16s4R. Lane M: Trackit 100bp ladder; Lane1: negative control (dH_2_O); Lane2: Reference strain (*C*. *jejuni subsp*. *jejuni* ATCC® 33291^™^; Lane3: T5; Lane4: T7; Lane5: T13.1; Lane6: T13.2; Lane7: T25.1; Lane8: T25.2; Lane9: T25.3; Lane10: T25.4; Lane11: T41.1; Lane12: T41.2; Lane13: T41.3; Lane14: T41.4; Lane15: T41.5.
(PDF)

S2 Fig Raw gel photograph of PCR using *Campylobacter* genus-specific primer-pair MD16S1-MD16S2. LaneM: Trackit 100bp ladder; Lane1: negative control (dH_2_O); Lane2: Reference strain (C. jejuni subsp. jejuni ATCC® 33291^™^; Lane3: T5; Lane4: T7; Lane5: T13.1; Lane6: T13.2; Lane7: T25.1; Lane8: T25.2; Lane9: T25.3; Lane10: T25.4; Lane11: T41.1; Lane12: T41.2; Lane13: T41.3; Lane14: T41.4; Lane15: T41.5.
(PDF)

S3 Fig Raw gel photograph of PCR using *A. butzleri specific* primer pair 16SArcobutzFw-16SArcobutzRv. LaneM: Trackit 100bp ladder; Lane1: negative control (dH_2_O); Lane2: Reference strain (*C*. *jejuni subsp*. *jejuni* ATCC® 33291^™^; Lane3: T5; Lane4: T7; Lane5: T13.1; Lane6: T13.2; Lane7: T25.1; Lane8: T25.2; Lane9: T25.3; Lane10: T25.4; Lane11: T41.1; Lane12: T41.2; Lane13: T41.3; Lane14: T41.4; Lane15: T41.5.
(PDF)

S4 Fig Raw gel photograph for ERIC profiles obtained for the 13 *A. butzleri* isolates. Lanes M: Trackit 100 bp DNA ladder; Lane1: negative control (dH_2_O); Lane2: Reference strain (*C*. *jejuni subsp*. *jejuni* ATCC® 33291^™^; Lane3: T5; Lane4: T7; Lane5: T13.1; Lane6: T13.2; Lane7: T25.1; Lane8: T25.2; Lane9: T25.3; Lane10: T25.4; Lane11: T41.1; Lane12: T41.2; Lane13: T41.3; Lane14: T41.4; Lane15: T41.5.
(PDF)

S5 Fig Raw photograph for dendrogram showing eight ERIC-types obtained for *A. butzleri* isolates. (PDF)
