# Supplementary material for: Molecular detection and genetic characterization of Arcobacter butzleri isolated from red-footed pet tortoises suspected for Campylobacter spp. from Grenada, West Indies
Source: PLoS One. 2020 Mar 16;15(3):e0230390. doi: 10.1371/journal.pone.0230390 (PMC7075591; doi:10.1371/journal.pone.0230390)
Supplement: S2 Fig — LaneM: Trackit 100bp ladder; Lane1: negative control (dH2O); Lane2: Reference strain (C. jejuni subsp. jejuni ATCC® 33291™; Lane3: T5; Lane4: T7; Lane5: T13.1; Lane6: T13.2; Lane7: T25.1; Lane8: T25.2; Lane9: T25.3; Lane10: T25.4; Lane11: T41.1; Lane12: T41.2; Lane13: T41.3; Lane14: T41.4; Lane15: T41.5. (PDF) [file pone.0230390.s002.pdf]

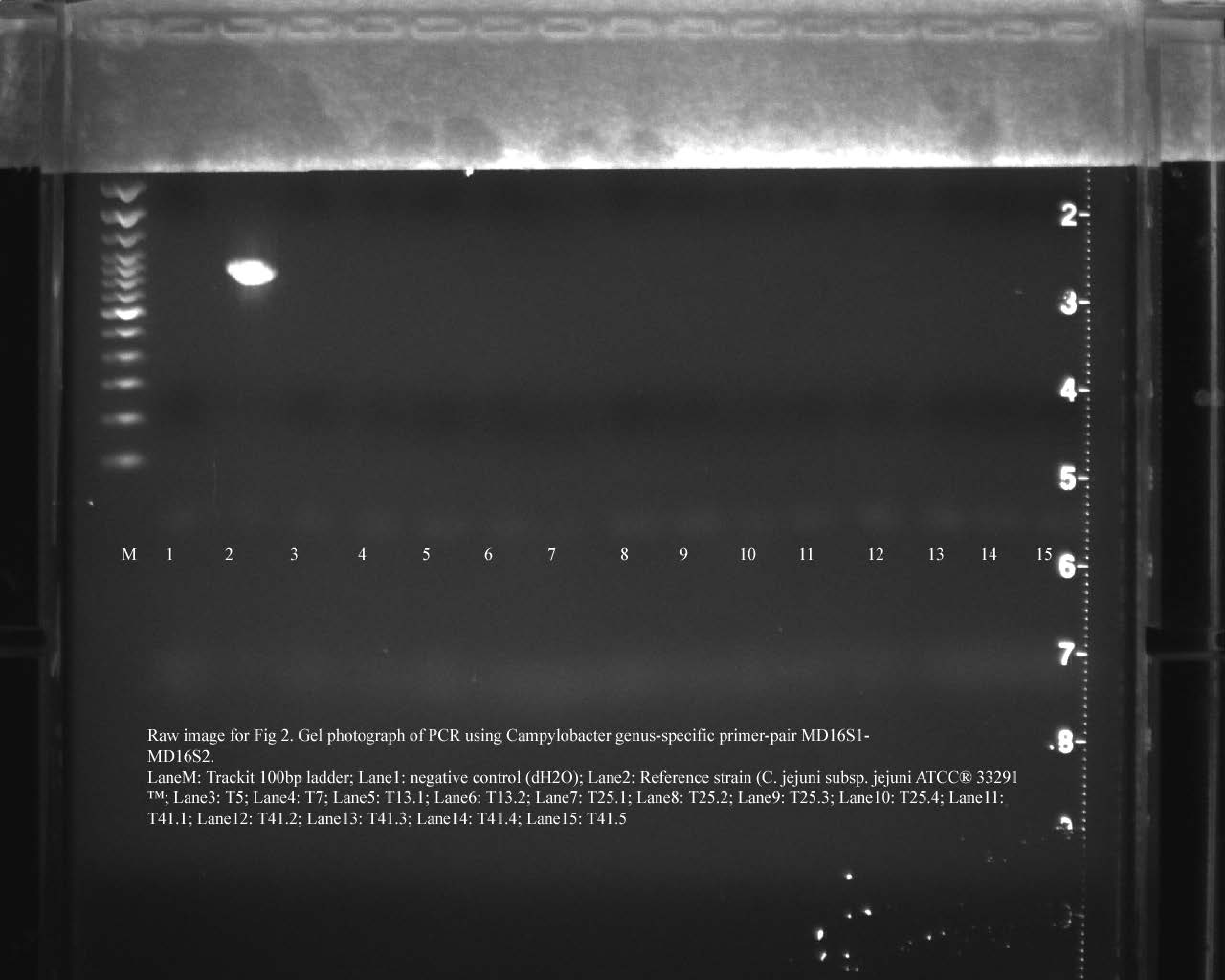

Raw image for Fig 2. Gel photograph of PCR using Campylobacter genus-specific primer-pair MD16S1-MD16S2.

LaneM: Trackit 100bp ladder; Lane1: negative control (dH2O); Lane2: Reference strain (C. jejuni subsp. jejuni ATCC® 33291 ™; Lane3: T5; Lane4: T7; Lane5: T13.1; Lane6: T13.2; Lane7: T25.1; Lane8: T25.2; Lane9: T25.3; Lane10: T25.4; Lane11: T41.1; Lane12: T41.2; Lane13: T41.3; Lane14: T41.4; Lane15: T41.5
