# Supplementary figures and images for: Molecular detection and genetic characterization of Arcobacter butzleri isolated from red-footed pet tortoises suspected for Campylobacter spp. from Grenada, West Indies
Source: PLoS One. 2020 Mar 16;15(3):e0230390. doi: 10.1371/journal.pone.0230390 (PMC7075591; doi:10.1371/journal.pone.0230390)

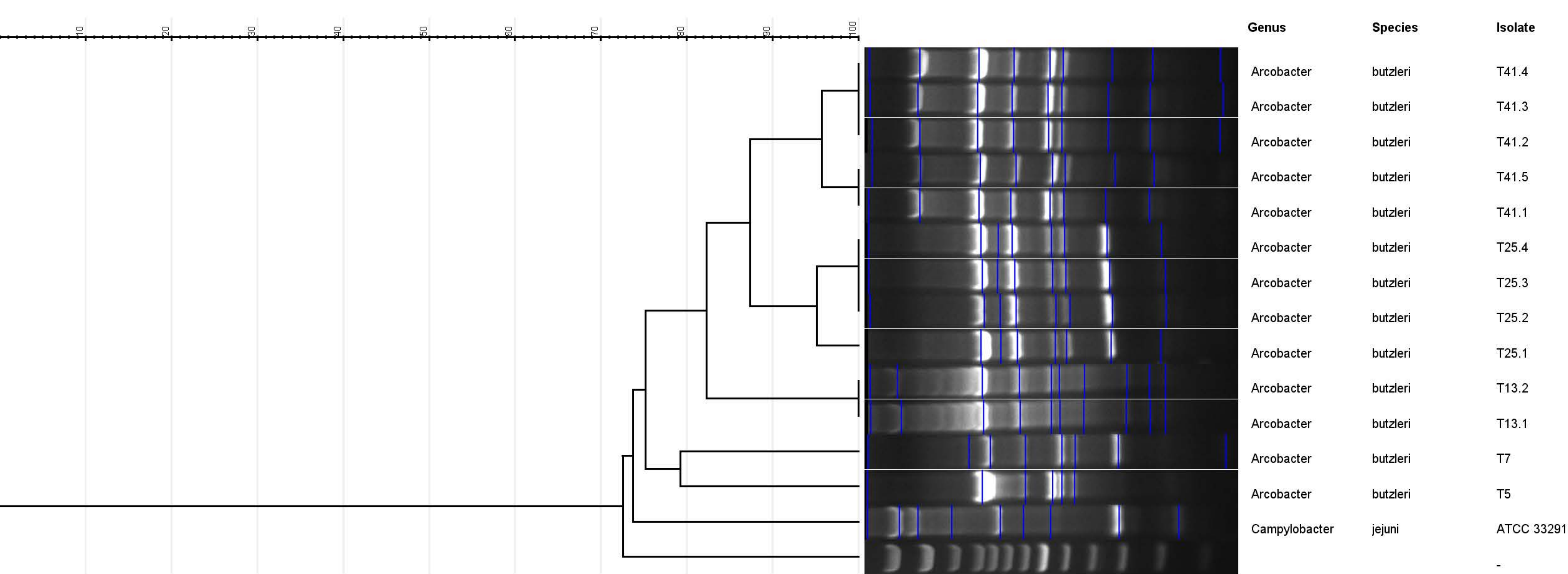

Raw image for Fig 5. Dendrogram showing eight ERIC-types obtained for *A. butzleri* isolates.

Supplement: S5 Fig — (PDF) [file pone.0230390.s005.pdf]
